# The Effect of COVID-19 and Its Control Measures on Sexual Satisfaction Among Married Couples in Kenya

**DOI:** 10.1016/j.esxm.2021.100354

**Published:** 2021-03-18

**Authors:** Joachim Osur, Edward Mugambi Ireri, Tammary Esho

**Affiliations:** School of Medical Sciences, Amref International University, Nairobi, Kenya; Amref Health Africa, End-FGM/C Centre of Excellence, ,; Amref International University, Nairobi, Kenya; Amref Health Africa, End-FGM/C Centre of Excellence, ,

**Keywords:** COVID-19 Curfew, COVID-19 Lockdown, Sexual Dissatisfaction, Sexual Satisfaction, Kenya, Married Couples

## Abstract

**Background:**

COVID-19 was first diagnosed in Kenya in March 2020 following which the government instituted control measures which could have affected people's sexual satisfaction and overall quality of life including restrictions in travels; ban on alcohol consumption and closure of bars; 9 pm to 5 am curfew; ban on political rallies, and closure of many workplaces with people being encouraged to work from home.

**Aim:**

The objective of this study was to determine how perceived and experienced sexual satisfaction changed with the advent of COVID-19 among heterosexual married individuals in Kenya.

**Methods:**

The study was a cross-sectional survey. Data was collected virtually using monkey survey from social groups. A total of 194 participants responded to the survey.

**Outcomes:**

The difference in overall sexual satisfaction as well as satisfaction with sex frequency; sex process; and time, place and ambience around sexual intercourse before and during COVID-19.

**Results:**

Most of the participants (73.4%) reported that they were satisfied with their marital sex before COVID-19 but the proportion of those reporting satisfaction dropped to 58.4% when they were asked about their experience during the COVID-19 pandemic. Among participants surveyed during the pandemic itself, therefore, 41.3% reported that they were currently sexually dissatisfied whereas just 26.6% reported that they were dissatisfied even prior to the pandemic. There was a significant difference in the overall distributions before and during COVID-19 (*χ²* = 38.86, *P*< .001).

**Clinical Translation:**

COVID-19 pandemic should be considered an etiology of sexual dissatisfaction and possibly sexual dysfunctions and COVID-19 control measures should incorporate ways of enhancing sexual well-being.

**Conclusion:**

There was perceived increase in dissatisfaction with sex which could be a pointer to the falling quality of life during COVID-19 pandemic especially among the most sexually active men aged 31–50 years living in places where COVID-19 control measures are being stringently implemented.

## Introduction

The Coronavirus disease 2019 (COVID-19) has infected millions of people across the world from the time the Director-General of the World Health Organization (WHO) declared it a global pandemic on March 11, 2020.^1^ The pandemic has brought with it a new normal way of life dictated by measures to control it.

COVID-19 control measures, especially lockdowns and curfews have caused couples to spend more time together. This together with other social and economic impacts of COVID-19 could have affected sexual satisfaction in couples. Previous studies have shown that the amount of time spent together may contribute to relationship confidence and satisfaction as well as improved intimacy.^2^ According to Milek et al^3^ shared time only improves intimacy if there is no intradyadic stress. It is not clear how COVID-19 and its control measures have changed these dynamics in relationships and impacted sexual satisfaction in Kenya.

Sexual satisfaction has two components: physical and emotional.^4^ Physical satisfaction refers to how fulfilling the latest sexual act was – the contextual factors around it such as how desirable the time of day when sex happened was; whether the place where it happened was acceptable; the ambience of the environment; as well as the desirability of the sexual process such as how enjoyable foreplay was and the extent to which intercourse was pleasurable. Emotional satisfaction is about happiness with the sex partner; the status of the relationship and the comfort in having sex with the person.

Previous studies have found that sexual satisfaction correlates with satisfaction with life generally so that if circumstances of life change, it also changes.^5^

At the same time, sexual satisfaction is a barometer of the health of the relationship. Where sex partners have an on-going conflict they are unlikely to be satisfied with sex.^6^ Generally, satisfactory older relationships tend to have satisfactory sexual outcomes. There is however gender difference in satisfaction with women tending to be less satisfied with sex early in relationships than later while men having more satisfaction the younger the relationship making age an important factor in sexual satisfaction.^7^

Fallis^6^ brings another twist to the subject of sexual satisfaction. In their study of sexual initiation, sexual frequency and sexual satisfaction in 101 couples, they concluded that perception of one's own sexual appeal is key in sexual satisfaction, confirming what has been known for long in sexual health about one's positive perception of own body image as being important in positive sexual experiences. In their study of social determinants of sexual satisfaction in Spain, Castellanos-Torres et al^8^ concludes that sexual satisfaction is 1.7 times higher among women who look after themselves and feel good about it.

Satisfaction is also affected by how one perceives and accepts their sexual partner. In one study in Iran women who were found to be sexually dissatisfied were also dissatisfied by their partners because of their social status, low-level jobs and low income.^9^

In summary, sexual satisfaction is a pointer to the health of the relationship, healthy sexuality, self-acceptance and self-pride, positive perception of sex partner and generally good quality of life. COVID-19 and restrictions to control it are likely to modify the factors that determine sexual satisfaction and either enhance or reduce it.

In a study done in Italy looking at the effect of COVID-19 on sexual functioning and quality of life among women living with their partners during the COVID-19 restrictions, it was found that sexual functioning and quality of life were significantly negatively affected.^10^ Authors related the effect to the psychological stress of the restrictions. A similar study done in China among men found increased rates of erectile dysfunction and quick ejaculation.^11^ Authors related the findings to increased anxiety and depression.

Another study done in Turkey and looking at female sexual functioning during COVID-19, however had mixed results. The study found women to have higher sexual desire and frequency during the pandemic and the overall sexual functioning index to have marginally reduced.^12^

The mixed findings of various studies on the scores for various domains of sexual functioning during COVID-19 show that COVID-19 related changes in life could affect satisfaction positively or negatively Arafat^13^ has summarized factors that have a positive influence as the ability of couples to spend more time together, decreased work burden, low social pressure and obligations and fewer opportunities to be involved in recreational activities. Negative factors influencing sex include an increase in interpersonal conflict, stress, lack of privacy, economic difficulties, and medical issues. The balance of the positive and negative influencers may result in overall satisfaction with sex during COVID-19.

This study investigated individuals’ sexual satisfaction before and after the COVID-19 pandemic was declared a public health emergency in Kenya. It measured couple's perception of their sexual satisfaction pre and during the pandemic so as to get an indication as to how life could have changed at individual and family levels during COVID-19. The COVID-19 restriction measures at the time included restrictions in travels within and out of the country; closure of schools; ban on alcohol consumption and closure of bars; 9 pm to 5 am curfew; ban on religious gatherings, weddings and funeral meetings; ban on political rallies, and closure of many workplaces with people being encouraged to work from home. The null hypothesis tested by this study is that COVID-19 and measures undertaken to control its spread did not change married couples’ perception of their sexual satisfaction. The study answered the question: did sexual satisfaction among married heterosexual couples in Kenya change when COVID pandemic hit the country and the government declared a state of emergency?

## Materials And Methods

This was a cross-sectional survey conducted from 15 to 30 of September 2020. Respondents of the study were resident in Kenya before and after the COVID-19 pandemic was declared as an emergency in the country. Purposive sampling was conducted among twenty virtual social groups of married couples with a total membership of about 3,000 drawn from across Kenya. Virtual groups of married couples are marriage support groups formed voluntarily by couples who want to improve their marriages. The groups normally have both wife and husband as members. Group members meet physically once in a while to learn about marriage, love and intimacy. Most of the activities are however virtual where they share reading materials, short videos and contribute their opinions on various subjects relating to relationships. We mapped out the groups through snowballing and found 20 of them. Administrators of the groups were contacted, and the study introduced to them including consent instructions. The group administrators then informed group members of the study and asked those interested to read through the consent form and if acceptable to them volunteer to participate anonymously. A link was provided at the end of the consent form for volunteers to click and access survey questions as consent to participate in the study. The survey targeted a sample number of 300 which is 10% of the target population.

A structured questionnaire adapted from the Index of Sexual Satisfaction^14^ was used (Appendix 1). The Index of Sexual Satisfaction, developed by Hudson in 1992, is a questionnaire designed to measure the degree of satisfaction in the sexual relationship between marital partners. Although not validated in Kenya, the tool has previously been validated and its reliability and validity found to be acceptable.^15^^,^^16^ Questions from the tool were revised to read in a culturally non-offensive way and secondly made short enough to allow for administration through the virtual platform. The research team jointly went through the questions to ensure that they met these two requirements.

The variables of focus in the questionnaire included the extent to which participants were happy with the frequency of sex; time and place where sex happened; the ambience around sex; and the whole process of sex including foreplay and penetrative sex. Participants were asked to compare their experiences before and during COVID-19. The last question was on overall satisfaction with sex before and during COVID-19 having considered all the variables. The study tool was digitalized using Google Survey Monkey https://www.surveymonkey.com/r/CKBG9FF. This made it possible for participants to use their phones or computers to respond to the survey. The digitalization of the data collection tool made it possible to overcome hurdles associated with these COVID-19 control measures. All that the study participants needed to do was to click on a link to access the survey on their devices. The link was shared with the virtual groups at the end of the consent form and with a covering message giving instructions on how to fill the questionnaire. Individual survey responses were automatically collected in the Monkey survey site. The responses were then exported and analyzed using SPSS version 25.^17^

The IP addresses identified participants’ location. This helped identify participants from Kenya and those from outside Kenya. The ones from outside Kenya were not included in the analysis. The IP addresses were also important in disaggregating geographical location within Kenya, indicating those from the capital City-Nairobi and those from other parts of Kenya. Gender age and the number of years in marriage were coded and managed as categorical data. Equally, ordinal and binary data were managed accordingly. Cross-tabulation was done to summarize the data. The chi-square statistic generated quantified the degree of the association on statistically significant interactions and vice versa. A strict threshold was adhered to while interpreting the significance of the chi-square statistic. The significant *P*-value at a 95% confidence interval, with 0% on the number of cells missing, was further subjected to analysis using binary logistic regression and the odds ratios determined.

The study was approved by the Ethics Review Committee of the Great Lakes University of Kisumu - a nationally accredited review board; and additionally by the National Commission for Science, Technology and Innovation.

## Results

### Descriptive Statistics

#### Details Of Study Participants

A total of 210 survey responses were received. The study had anticipated obtaining 300 survey responses based on 10% of the total population of interest, that is, twenty virtual groups of married couples with a membership of about 3000. This meant that 70% of the anticipated sample size was achieved. Equally, 16 survey responses were excluded from the study because their IP addresses indicated that the respondents were outside Kenya and thus the sample size reduced to 194 survey responses.

The majority of respondents were from Nairobi city (89.2%, *n* = 173). Males made 60.8% of participants (*n* = 118). Those of the age bracket of 41–50 years were the majority at 41.8% followed by those at 31–40 years; 18–30 years; and over 50 years olds at 35.3%; 12.4% and 10.8%, respectively. Most participants had been in the marriage for 11–20 years followed by 3–10 years at 38.1% and 30.9%, respectively.

#### Satisfaction With Marital Sex Before And During Covid-19

Participants responded to questions on various aspects of their sexual experiences before and after COVID-19 to determine the basis of their overall satisfaction or dissatisfaction with sex before and during COVID-19. The aspects included satisfaction with frequency; process; time of day; place; and ambience around sex. Table 1 summarizes responses to these variables.

**Table 1:** Participant's sexual experiences before and during COVID-19

Desired frequency of sex										
	Overall satisfaction with sex	Want a lot more often		Want a little more often	Want less often	Happy with the frequency of sex				
Before COVID-19	Dissatisfied (26.6%)	20 (11.6%)		22 (12.7%)	1 (0.6%)	3 (1.7%)			*χ²* = 21.06, *P*<.001	
	Satisfied (73.4%)	33 (19.1%)		35 (20.2%)	6 (3.5%)	53 (30.6%)				
During COVID-19	Dissatisfied (41.6%)	36 (20.8%)		24 (13.9%)	7 (4%)	5 (2.9%)		*χ²* = 37.92, *P*<.001		
	Satisfied (58.4%)	19 (11%)		21 (12.1%)	14 (8.1%)	47 (27.2%)				
Acceptability of the sexual process										
	Overall satisfaction with sex	Rarely or Never	Once in a While	Half of the time	Often	All the time				
Before COVID-19	Dissatisfied (26.6%)	12 (6.9%)	15 (8.7%)	13 (7.5%)	6 (3.5%)	0 (0%)	*χ²* = 35.71, *P*<.001			
	Satisfied (73.4%)	7 (4%)	17 (9.8%)	31 (17.9%)	42 (24.3%)	30 (17.3%)				
During COVID-19	Dissatisfied (41.7)	29 (16.8%)	22 (12.7%)	14 (8.1%)	5 (2.9%)	2 (1.2%)	*χ²* = 53.09, *P*<.001			
	Satisfied (58.3%)	6 (3.5%)	15 (8.7%)	26 (15%)	31 (17.9%)	23 (13.3%)				
Having sex at a preferred time of the day										
	Overall satisfaction with sex	Rarely or Never	Once in a While	Half of the time	Often	All the time				
Before COVID-19	Dissatisfied (26.6)	15 (8.7%)	13 (7.5%)	11 (6.4%)	7 (4%)		*χ²* = 34.86, *P*<.001			
	Satisfied (73.4%)	6 (3.5%)	22 (12.7%)	41 (23.7%)	35 (20.2%)	23 (13.3%)				
During COVID-19	Dissatisfied (41.6)	33 (19.1%)	21 (12.1%)	15 (8.7%)	3 (1.7%)		*χ²* = 58.68, *P*<.001			
	Satisfied (58.4%)	6 (3.5%)	17 (9.8%)	35 (20.2%)	43 (24.9%)					
Having sex at a place of choice										
Before COVID-19	Overall satisfaction with sex	Rarely or Never	Once in a While	Sometimes	Often	All the time!				
	Dissatisfied (26.6%)	24 (13.9%)	11 (6.4%)	6 (3.5%)	3 (1.7%)	2 (1.2%)	*χ²* = 22.87, *P* <.001			
	Satisfied (73.4%)	25 (14.5%)	23 (13.3%)	40 (23.1%)	28 (16.2%)	11 (6.4%)				
During COVID-19	Dissatisfied (41.6%)	48 (27.7%)	13 (7.5%)	5 (2.9%)	4 (2.3%)	2 (1.2%)	*χ²* = 41.36, *P*<.001			
	Satisfied (58.4%)	21 (12.1%)	22 (12.7%)	29 (16.8%)	22 (12.7%)	7 (4%)				
The desirability of the ambience of the place where sex happened										
Before COVID-19	Overall satisfaction with sex	Rarely or Never	Once in a While	Sometimes	Often	All the time!				
	Dissatisfied (26.6)	20 (11.6%)	12 (6.9%)	5 (2.9%)	9 (5.2%)	0 (0%)	*χ²* = 31.56, *P*<.001			
	Satisfied (73.4%)	14 (8.1%)	21 (12.1%)	36 (20.8%)	40 (23.1%)	16 (9.2%)				
During COVID-19	Dissatisfied (41.6)	39 (22.5%)	14 (8.1%)	9 (5.2%)	10 (5.8%)	0 (0%)	*χ²* = 36.63, *P*<.001			
	Satisfied (58.4%)	16 (9.2%)	16 (9.2%)	32 (18.5%)	27 (15.6%)	10 (5.8%)				

#### Overall Satisfaction

The majority of participants, 73.4%, were satisfied with their marital sex before COVID-19 but this fell to 58.4% when COVID-19 and related control measures came into place. The proportion of those dissatisfied therefore rose from 26.6%.001).

#### Contributors To The Significant Rise In Dissatisfaction With Marital Sex

Frequency of sex was the main contributor to dissatisfaction with marital sex. Before COVID-19 pandemic 63.6% of participants wanted sex more frequently and only 4.1% wanted it less often. Those who wanted sex more often however dropped to 57.8% during COVID-19, those wanting sex less often rising to 21.1%. Even though there was a significant difference in the distribution of those satisfied and those dissatisfied with the frequency of sex before COVID-19, the statistical difference grew stronger during COVID-19 with *χ²* rising from 21.06 to 37.92, in both cases *P*< .001. There was an overall increase in the number of those dissatisfied with sex frequency irrespective of whether they wanted the frequency to increase, go down or remain the same.

The sexual process, including foreplay, sexual position and the speed of sex was also a contributor to dissatisfaction. Before COVID-19 pandemic, 29.4% of participants were rarely, never or only had the desired process once in a while. This proportion increased to 41.7% during the pandemic. At the same time, those who had the desired process often or all the time decreased from 45.1% to 35.3%. While there was a significant difference in distribution among those satisfied and those dissatisfied with marital sex before COVID-19, the situation worsened during COVID-19 among the distributions (*χ²* changing from 35.71 to 53.09; *P*< .001 in both cases).

On the desirability of time of day when sex happened, 32.4% of participants rarely, never or only had sex at the desired time of day once in a while. This proportion rose to 44.5% during COVID-19. Before COVID-19 37.5% of participants had sex at the desired time of day often or all the time but this dropped to 26.6% during COVID-19. The trend was to have more people dissatisfied with the time of day when sex happened during COVID-19 pandemic (*P* < .001).

Similar trends of significantly more participants being dissatisfied were noted with the desired place where sex happened as well as the general aura (ambience) around sex. In all cases, there was increased discordance in what was desired versus what was experienced before and during COVID-19. Table 1 summarizes these trends.

When disaggregated by age, the only significant difference noted was on the time of day when sex happened in the 41–50-year olds before and during COVID-19, more people in this age group were more dissatisfied with the timing of sex during COVID-19. Disaggregating the data by geography was revealing. There was virtually no significant difference in any of the distributions before and during COVID-19 for populations from outside of the city of Nairobi although only a small number of 21 respondents were from outside Nairobi. At the same time, all variables showed significant difference with more dissatisfaction during COVID-19 for Nairobi populations. It is notable that Nairobi was the epicenter of COVID-19 during this study and that enforcement of COVID-19 control restrictions were more stringent in Nairobi than in other parts of Kenya.

A summary of significant finds on binary logistics regression is presented in Table 2 . The findings show that the main factor resulting in the difference between those satisfied and those not satisfied with marital sex before COVID-19 was satisfaction with the process of sex among females aged 31–40 years including how foreplay, sex position and speed of sex (fast or slow) was. Overall dissatisfaction with marital sex during COVID-19, however, resulted significantly from men aged 31–40 years old being dissatisfied with sex during this period compared to before COVID-19. There was also a significant difference in the different levels of satisfaction in this group during the period. Considering the duration of the marriage, men in the bracket of 3–10 and 10–20 years of marriage and aged 31–40 and 41 to 5–0 years respectively made a significant contribution to the dissatisfaction with sex seen during COVID-19 based on their change in the overall level of satisfaction with sex from before to during the pandemic.

**Table 2:** Summary of significant findings on binary logistics regression

Period	*Age*	*Sex*	*Characteristic*	*B*	*S.E.*	*P-value*	*Exp(B)*	
B	31–40	Female	Process of sex before COVID-19	1.509	0.754	.045	4.523	
			Constant	−4.587	2.332	.049	0.01	
	*Age*	*Sex*	*Characteristic*	*B*	S.E.	*P*-*value*	*Exp(B)*	
D	31–40	Male	Overall sexual satisfaction with your spouse during COVID	2.011	0.724	.006	7.468	
			Constant	−4.454	2.5	.075	0.012	
		Male	Level of overall sexual satisfaction during COVID-19	1.796	0.578	.002	6.024	
			Constant	−4.769	2.277	.036	0.008	
	*Age*	*Sex*	*Years in marriage*	*Characteristic*	*B*	*S.E.*	*P*-*value*	*Exp(B)*
D	31–40	Male	3–10 years	Level of overall sexual satisfaction	2.55	1.223	.037	12.809
				Constant	−5.02	3.423	.142	0.007
	41–50	Male	11–20 years	Level of overall sexual satisfaction	1.621	.567	.004	5.059
				Constant	−3.774	2.203	.087	0.023

Notes: B = Before COVID-19 pandemic, D = During COVID-19 lockdown. This table shows 2-way and 3-way interactions. The findings presented are only significant for Nairobi County.

## Discussion

This study showed a pattern of increasing dissatisfaction with sex during COVID-19 lockdown compared to before COVID-19 pandemic in Kenya among married couples with overall satisfaction falling from 73.4% to 58.4% (*P*< .001). The findings are in concurrence with several study findings across the world such as those of^18^ and^19^.

Looking at possible contributors to the increasing dissatisfaction with marital sex during COVID-19, this study showed increasing dissatisfaction with sexual frequency, process, time of day when sex happened as well as the place and aura around sex. This means that dissatisfaction was not synonymous with the desire for less sex. Neither was it synonymous with desire for more. It was also not synonymous with the desire to maintain existing frequency. This finding is different from the over-sweeping finding in some studies that COVID-19 had sex frequency go down and in others that it made frequency go up. Irrespective of how frequency changed, our finding is that satisfaction reduced. Previous studies^20^^,^^21^ have shown a reduction in sexual desire and frequency during COVID-19 which may point towards a negative change in satisfaction. A study by^12^ however had opposite findings to the other studies and showed increased sex desire and frequency during COVID-19. Irrespective of how the frequency changed, however, this study shows an overall reduction in satisfaction.

COVID-19 and related restrictions have led to a change in living circumstances with increased family supervision, less personal freedom and privacy and deteriorating mental health.^18^^,^^20^ These factors could have been enhanced in Nairobi by having children at home because schools were closed, working from home, restrictions in public transportation and stringent enforcement of all other COVID-19 control restrictions in Nairobi compared to other parts of Kenya. The result is that the personal COVID-19 experience could have led to a change in sexual behavior including the frequency, process, time of day and the place and ambience of where sex happened. The overall effect was increased dissatisfaction with sex and, as shown in the regression analysis, males aged 31–40 years and being in a marriage for less than 20 years exhibited the most dissatisfaction, possibly because this is the time when sexual activity is highest. The same group could be suffering the most reduction in quality of life with increased psychological stress since sexual satisfaction is a pointer to the overall quality of life.^5^

This study has important clinical implications for sexual health and sexual medicine in the era of COVID-19. Assessment of patients for sexual functioning must now include changes in life circumstances brought about by COVID-19 and how the changed circumstances are affecting sexual routines. It must include how life circumstances are impacting psychological wellbeing and mental health and the resultant effect on sexual functioning. Clinicians, epidemiologists and researchers in sexual health and sexual medicine need to revise standard tools that have hitherto been used to incorporate these new COVID-19 realities.

The findings of this study should further inform the design of COVID-19 control programs. Unless control measures are comprehensive, they may leave people with others which are equally harmful to their medical and social wellbeing. Sexual health should be included in making COVID-19 interventions comprehensive.

A lot more studies are however needed to build evidence on the positive and negative influencers for sexual satisfaction in the era of COVID-19. This is especially because the disease is dynamic and presents new challenges and realities each day which impact sexuality both positively and negatively. For example, several studies have been done on couple's sexuality during COVID-19 under the western context. Unfortunately, most of these studies are not in Africa and so we do not know whether Africans have experienced COVID-19 the same way as in other continents. In Kenya where the study was done, the authors of this study have not come across similar studies. We believe that knowledge from Africa will enrich the already available information from other parts of the world and that this diversity of knowledge will help us understand better the effect of COVID-19 on sexuality. We also acknowledge that Africa has had a delay in the escalation of the COVID-19 epidemic and that studies similar to this started earlier in countries where COVID-19 hit earlier. Along the way, as we got the study approved by the ethical board, results from other countries were trickling in. Our study, therefore, seems to be coming late in the day but it is also a reflection of how the pandemic has progressed across the world.

## Conclusion

COVID-19 experience has had an impact on sexual lives of individuals leading to increasing discordance between what married people want and what they are experiencing with sex frequency, sex process, and time, place and aura around sex. This discordance has contributed to increasing dissatisfaction with sex which could be a pointer to the falling quality of life, especially among the most sexually active men aged 31–50 years living in places where COVID-19 control measures are being stringently implemented. In this study, the response rate for male was high compared with women. This could be attributed to some cultural factors We will need to see trends in similar studies in Africa in future to conclude on this. Since this particular study did not have a qualitative arm we were unable to have a factual answer on the role of the culture on sexual satisfaction under the African context.

## Study Limitations

The study focused on couples who are in virtual social groups, have a smart phone or computer and can access internet. This sampling methodology excluded many couples, weakening the generalizability of the study. Participants of the social groups are also of high literacy and not an exact representation of all Kenyan couples. The study had anticipated sample size of 300 but ended up with 197 respondents. This has weakened the generalizability of the findings. We do understand how difficult it is for people to give information on their sexuality which could have contributed to this lower sample size. The study is also limited by recall bias as people had to fill in their historical data on sexual satisfaction prior to COVID-19. Another limitation is that people not in marital relationships and those in homosexual relationships could have experienced COVID-19 differently and were not included. The study did not collect qualitative information to explain quantitative findings. This is quite limiting and is a gap that requires further studies in future. The study was limited to sexual satisfaction with heterosexual partner sex and did not explore solo sex or homosexual sex which can co-exist with heterosexual sex in long term relationships.

## Ethical Considerations

The study was approved by the Great Lakes University Ethical Review Committee and National Commission for Science, Technology and Innovation (NACOSTI). Inherent risks to the study participants were assessed and found to be negligible – participant autonomy and privacy were maintained. It was not possible to link information shared to the source. Further, there was no health risks associated with the study. The study only carries benefits since the information obtained can be used to improve sexual health and psychological services in the current and any future lockdowns. On the issue of consent, a cover email was written accompanying request for participation and explaining that the study is voluntary and that by filling the questionnaire it was taken that participants had consented to it without coercion.

## Statement Of Authorship

Joachim Osur: Conceptualization, Methodology, Investigation, Validation, Writing – Original Draft, Writing -Review & Editing, Funding Acquisition, Resources, Supervision; Edward Mugambi Ireri: Methodology, Validation, Formal Analysis, Writing – Original Draft, Writing -Review & Editing, Visualisation, Resources; Tammary Esho: Methodology, Investigation, Validation, Writing -Review & Editing, Funding Acquisition, Resources, Supervision, Project Administration.

## Notes

*Funding* None.

## Appendix 1: The Sexual Satisfaction Questionnaire

1. Your age
  ○ A 18 – 30
  ○ B 31 – 40
  ○ C 41 – 50
  ○ D 51 – 60
  ○ E Above 60
2. Your sex
  ○ A Male
  ○ B Female
  ○ C Intersex
  ○ D Others
3. Number of years in your marriage/relationship
  ○ A 0 – 2 Years
  ○ B 3 – 10 Years
  ○ C 11 – 20 Years
  ○ D Above 20 Years
4. How was your level of overall sexual satisfaction with your spouse before the Corona pandemic?
  ○ Highly Satisfied!
  ○ Very Satisfied!
  ○ I'm Satisfied.
  ○ Could be better.
  ○ No satisfaction.
5. How is your level of overall sexual satisfaction with your spouse during the Corona lockdown/curfew?
  ○ Highly Satisfied!
  ○ Very Satisfied!
  ○ I'm Satisfied.
  ○ Could be better.
  ○ No satisfaction.
6. Before the Corona pandemic, I always wished my spouse and I would have sex:
  ○ A lot more often!
  ○ A lot less often!
  ○ A little more often
  ○ A little less often
  ○ We have enough sex as is!
7. During this corona pandemic, I wish my spouse and I would have sex:
  ○ A lot more often!
  ○ A lot less often!
  ○ A little more often
  ○ A little less often
  ○ We have enough sex as is!
8. Before the Corona pandemic my partner and I had sex the way I wanted (foreplay, position, fast, slow):
  ○ All the time!
  ○ Often
  ○ Sometimes
  ○ Once in a while...
  ○ Rarely or Never!
9. During this Corona Lockdown/curfew my partner and I have sex the way I want (foreplay, position, fast, slow):
  ○ All the time!
  ○ Often
  ○ Sometimes
  ○ Once in a while...
  ○ Rarely or Never!
10. Before the Corona pandemic my mate and I had sex WHEN I wanted to (at night before bed, in the morning, at noon, etc.):
  ○ All the time!
  ○ Often
  ○ Sometimes
  ○ Once in a while...
  ○ Rarely or Never!
11. During the Coronal lockdown/curfew my mate and I have sex WHEN I want to (at night before bed, in the morning, at noon, etc.):
  ○ All the time!
  ○ Often
  ○ Sometimes
  ○ Once in a while...
  ○ Rarely or Never!
12. Before the Corona pandemic my spouse and I had sex WHERE I wanted to (on the bed, on the kitchen counter, in the tub, on a chair):
  ○ All the time!
  ○ Often
  ○ Sometimes
  ○ Once in a while...
  ○ Rarely or Never!
13. During the Corona lockdown/curfew my spouse and I have sex WHERE I want to (on the bed, on the kitchen counter, in the tub, on a chair):
  ○ All the time!
  ○ Often
  ○ Sometimes
  ○ Once in a while...
  ○ Rarely or Never!
14. Before the Corona pandemic my partner and I had sex in the kind of environment that I liked (lights on, lights off, dimmed lights, romantic setting, quiet, outdoors, in the car, very private area, where we can get caught):
  ○ All the time!
  ○ Often
  ○ Sometimes
  ○ Once in a while...
  ○ Rarely or Never!
15. During the Corona lockdown/curfew my partner and I have sex in the kind of environment that I like (lights on, lights off, dimmed lights, romantic setting, quiet, outdoors, in the car, very private area, where we can get caught):
  ○ All the time!
  ○ Often
  ○ Sometimes
  ○ Once in a while...
  ○ Rarely or Never!
16. Before the Corona problem my spouse was sexually satisfied with me:
  ○ All the time!
  ○ Most of the time
  ○ Sometimes
  ○ Once in a while...
  ○ Rarely or Never!
17. During the Corona lockdown/curfew my spouse is sexually satisfied with me:
  ○ All the time!
  ○ Most of the time
  ○ Sometimes
  ○ Once in a while...
  ○ Rarely or Never!
18. Before the Corona pandemic, I felt that our sex life was improving. (Do you agree with this?)
  ○ Strongly agree
  ○ I agree
  ○ Somewhat agree
  ○ Somewhat disagree
  ○ Strongly disagree
19. During this Corona lockdown/curfew, I feel that our sex life is improving. (Do you agree with this?)
  ○ Strongly agree
  ○ I agree
  ○ Somewhat agree
  ○ Somewhat disagree
  ○ Strongly disagree
20. Before the Corona pandemic my spouse fulfilled my greatest sexual desire:
  ○ Yes and often!
  ○ Yes, but not often.
  ○ Yes, once or twice.
  ○ No, but I think he/she will eventually do so.
  ○ No and I don't think it will ever happen!
21. During this Corona lockdown/curfew my spouse fulfils my greatest sexual desire:
  ○ Yes and often!
  ○ Yes, but not often.
  ○ Yes, once or twice.
  ○ No, but I think he/she will eventually do so.
  ○ No and I don't think it will ever happen!
22. During this Corona lockdown/curfew, how sexually attractive do you find your spouse? (5 being highest and 1 being lowest)
  ○ 5
  ○ 4
  ○ 3
  ○ 2
  ○ 1
23. Before the Corona pandemic how sexually attractive did you find your spouse? (5 being highest and 1 being lowest)
  ○ 5
  ○ 4
  ○ 3
  ○ 2
  ○ 1
24. Before the Corona pandemic how sexually attractive did you think you were to your spouse?
  ○ 5
  ○ 4
  ○ 3
  ○ 2
  ○ 1
25. During this Corona lockdown/curfew, how sexually attractive do you think you are to your spouse?
  ○ 5
  ○ 4
  ○ 3
  ○ 2
  ○ 1
26. Were you satisfied with your marital sex life before the Corona pandemic?
  ○ Yes
  ○ No
27. Are you satisfied with your marital sex life during this Corona lockdown/curfew?
  ○ Yes
  ○ No
